# Meetings of Societies

**Published:** 1928

**Authors:** 


### Meetings of Societies.

### Bristol and Bath Surgical Club.

The twelfth meeting of the Bristol and Bath Surgical Club was held in London on Friday and Saturday, December 9th and 10th, 1927, when ten members attended.

Friday afternoon was spent at King's College, and the session was opened by an address on pyelograms by Sir John Thomson-Walker, who showed the pitfalls which may entrap the unwary. He also demonstrated the value of serial pyelography for the smaller hydronephroses. After this we had the keen pleasure of seeing him operate—for a renal pelvic stone in an undeliverable kidney, and for a lumbar ureteric calculus. Also he convincingly exemplified his method of open prostatectomy, especially in a case of tuberculosis of prostate and seminal vesicles where the diseased organs were dissected *en bloc* by the transvesical route. Anæsthesia was obtained by a preliminary spinal injection of stovaine followed by a small dose of general anæsthetic.

After the business meeting at the Langham Hotel, the Club dined with Sir John Thomson-Walker and Sir Percy Sargent. The latter gave it as his opinion that the surgery of any special part of the body is best developed by general surgeons bringing to it the wealth of a wide general experience rather than by early specialisation.

Saturday, 9 a.m., found us at the National Hospital for Nervous Diseases, to watch Sir Percy Sargent operate. He first removed a tangerine-sized endothelioma of the left parietal region involving bone and dura. Hemorrhage was arrested with meticulous care. Next came the removal of a large pituitary adenoma by the transfrontal route. Incision and aspiration of the contents were carried out by a suction tube connected with the anæsthetic motor. Anæsthesia by the intratracheal method seemed almost automatic after the preliminary induction. An hour in the lecture theatre with slides, specimens and patients closed a pleasant and highly instructive meeting.

